# The Clinical Application of Mealtime Whey Protein for the Treatment of Postprandial Hyperglycaemia for People With Type 2 Diabetes: A Long Whey to Go

**DOI:** 10.3389/fnut.2020.587843

**Published:** 2020-10-20

**Authors:** Kieran Smith, Kelly A. Bowden Davies, Emma J. Stevenson, Daniel J. West

**Affiliations:** ^1^Population Health Sciences Institute, Newcastle University, Newcastle upon Tyne, United Kingdom; ^2^Department of Sport and Exercise Sciences, Manchester Metropolitan University, Manchester, United Kingdom

**Keywords:** type 2 diabetes, postprandial hyperglycaemia, whey protein, second meal effect, pre-load, postprandial glycaemic control

## Abstract

Mitigating postprandial hyperglycaemic excursions may be effective in not only enhancing glycaemic control for people with type 2 diabetes but also reducing the onset of diabetes-related complications. However, there are growing concerns over the long-term efficacy of anti-hyperglycaemic pharmacotherapies, which coupled with their rising financial costs, underlines the need for further non-pharmaceutical treatments to regulate postprandial glycaemic excursions. One promising strategy that acutely improves postprandial glycaemia for people with type 2 diabetes is through the provision of mealtime whey protein, owing to the slowing of gastric emptying and increased secretion of insulin and the incretin peptides. The magnitude of this effect appears greater when whey protein is consumed before, rather than with, a meal. Herein, this dietary tool may offer a simple and inexpensive strategy in the management of postprandial hyperglycaemia for people with type 2 diabetes. However, there are insufficient long-term studies that have investigated the use of mealtime whey protein as a treatment option for individuals with type 2 diabetes. The methodological approaches applied in acute studies and outcomes reported may also not portray what is achievable long-term in practice. Therefore, studies are needed to refine the application of this mealtime strategy to maximize its clinical potential to treat hyperglycaemia and to apply these long-term to address key components of successful diabetes care. This review discusses evidence surrounding the provision of mealtime whey protein to treat postprandial hyperglycaemia in individuals with type 2 diabetes and highlights areas to help facilitate its clinical application.

## Introduction

For people with type 2 diabetes [T2D], the regulation of postprandial glycaemia [PPG] is critical to achieving optimal glycaemic control (1, 2), which may mitigate complications associated with T2D and hyperglycaemia (3–7). Indeed, PPG excursions are associated with deleterious effects on the vasculature (8), and independently predict the onset of microvascular complications (9) and the incidence of future cardiovascular events (10, 11). The targeted treatment of PPG is, however, secondary, only occurring when glycaemic targets have not been met (12). Yet, meeting clinically desirable HbA_1c_ values does not always preclude postprandial hyperglycaemic excursions (13). Accordingly, treatment strategies should be employed to focus not only on reducing HbA_1c_ but controlling PPG.

Several pharmacotherapies are effective in treating hyperglycaemia (12). However, there is uncertainty regarding their efficacy long-term (14). There are also increasing concerns with the safety and side effects of commonly prescribed agents, including thiazolidinediones, sulfonylureas and prandial insulins (15), which coupled with their rising costs (16), underscores the need for non-pharmaceutical strategies for glycaemic management.

Accumulating evidence suggests that nutrient *pre-loading* can profoundly improve PPG for people with T2D (17–20). Consuming a macronutrient at a fixed interval before a meal primes gluco-regulatory milieu allowing for the efficient sequestration of glucose from the subsequent feed (18, 19, 21). The efficacy of this response is, however, dependent on the nutrient composition of the pre-load (22) with a body of work supporting the use of dietary protein (17, 23), and in particular, whey protein [WP] as an effective strategy (19, 24).

In this review, we appraise recent evidence surrounding the provision of mealtime WP on PPG regulation in people with T2D and proceed to discuss experimental shortcomings surrounding this nutritional strategy and their implications for future research and clinical practice.

## Whey Protein and A Postprandial Glycaemia Reducing Milieu

**Figure 1:**
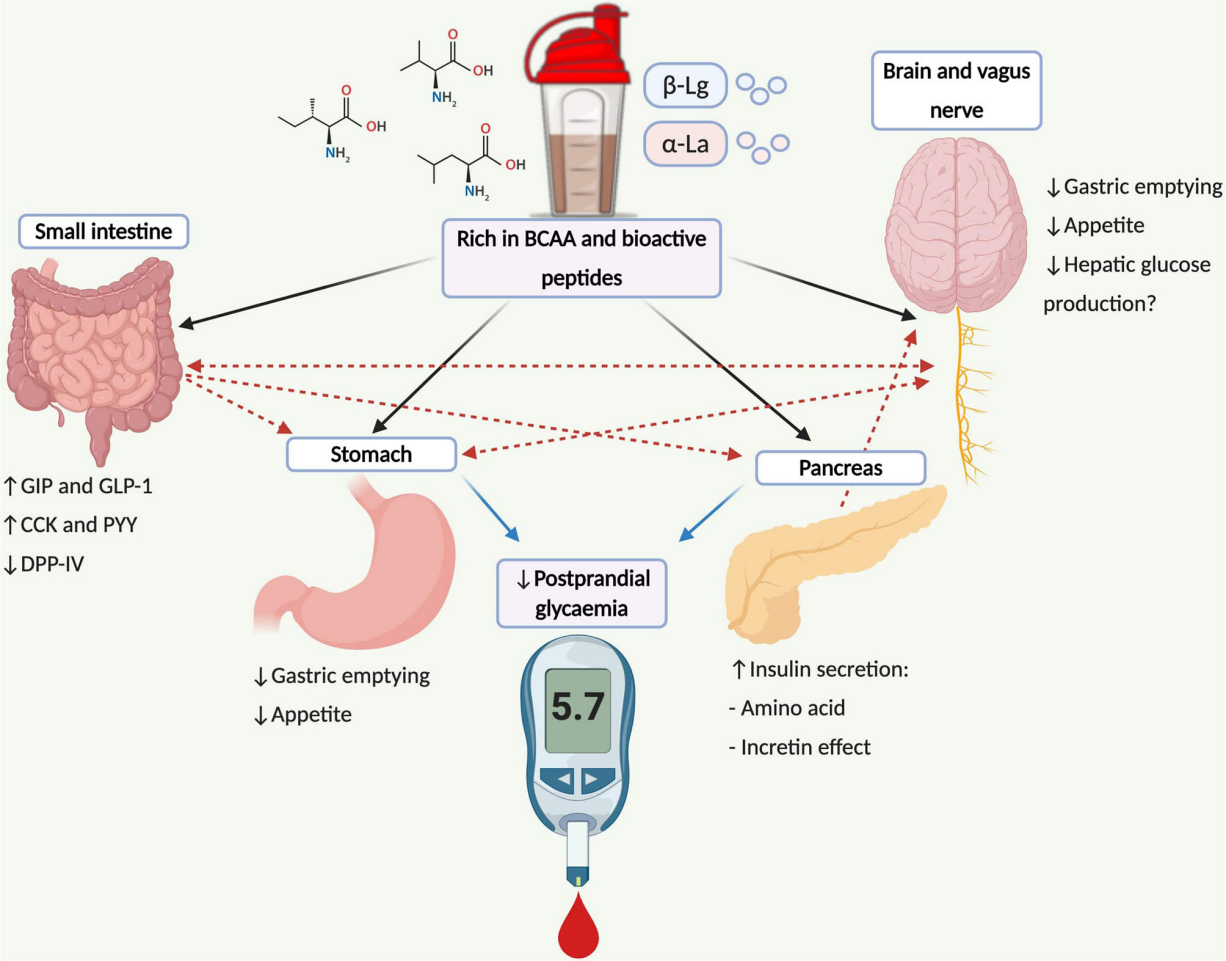
Schematic illustration depicting mechanisms and pathways associated with postprandial glucose regulation following WP consumption and its potential application for type 2 diabetes management. WP is rich in BCAA (leucine, isoleucine and valine) and bioactive peptides (α-La and β-Lg) that stimulate the secretion of the incretin peptides and insulin from pancreatic β-cells. Incretin peptides, particularly GLP-1, stimulate β-cell activity, augmenting the release of insulin, where GLP-1 also regulates the rate of gastric emptying mediated through vagal afferents that convey information to the brainstem. Further gut peptides including CCK and PYY are stimulated following WP ingestion, which also delay the rate of gastric emptying and regulate the gastrointestinal transit of food via central-related mechanisms. Bioactive peptides residing WP may also inhibit DPP-IV activity, increasing the concentrations of intact incretin moieties. Insulin can also cross the blood brain barrier within the central nervous system, which may suppress appetite and regulate hepatic glucose production via the brain-liver axis. Dashed lines represent combined influences. BCAA, branch chain amino acids; CCK, cholecystokinin; DPP-IV, dipeptidyl-peptidase IV; GIP, glucose-dependent insulinotropic peptide; GLP-1, glucagon-like peptide-1; PYY, peptide tyrosine tyrosine; α-La, α-lactalbumin; β-Lg, β-lactoglobulin.

WP is rich in branched-chain amino acids [BCAA] and bioactive peptides that augment several interconnected mechanisms associated with PPG regulation (Figure 1). Briefly, these include: amino-acid induced insulinemia (25, 26); augmentation of the incretin effect (26, 27); and suppression of dipeptidyl peptidase IV [DPP-IV] activity (28–30)—although work within humans is unclear (31). Additionally, WP delays the rate of gastric emptying (19, 32–34)–*likely* mediated by glucagon-like peptide 1 [GLP-1]-related mechanisms (35, 36)–purported as a central mechanism associated with improved PPG handling (37). The latter mechanism is of importance for individuals with controlled T2D who demonstrate accelerated gastric emptying rates (38, 39). The regulation of gastric emptying following mealtime WP may also be of significance for patients with diminished β-cell function (40), where improvements in PPG have been reported following treatments that slow gastric emptying in absence of increased insulin concentrations (40, 41).

## Mealtime Whey Protein as a Non-Pharmacological Approach To Treat Postprandial Glycaemia

Several lines of evidence demonstrates the capacity of WP to regulate PPG in patient (19, 31–34, 42, 43) and non-patient (44–46) populations. A summary of acute randomized-control trials utilizing mealtime WP to control PPG in individuals with T2D is presented in Table 1. Yet, the clinical impact of this inherently simple strategy remains unclear. Although Trico et al. boldly postulate that a protein-rich nutrient pre-load may offer similar effects to what can be achieved with available anti-hyperglycaemic agents (17), as previously proposed by Jakubowicz et al. (31), objective evidence supporting such claims is limited.

**Table 1:** A summary of acute, randomized-control, crossover trials that have utilized mealtime WP to regulate postprandial glycaemic excursions in people with T2D.

**References**	**Studied cohort**	**Design**	**Treatment**	**Comparator**	**Findings**	**Key messages**
Bjørnshave et al. (47)	Metformin-controlled T2D (*n* = 12; 3F/9M) - Age: 62.9y [57–68.8] - HbA_1c_: 50 mmol**·**mol [range: 46–52] - Dx: Not reported. Age-weight matched controls (*n* = 12)	- Randomized, crossover design. - Two visits separated by 7d. - Treatments were consumed before (15 min) or with a high-fat breakfast meal (932 kcal: 40 g CHO) + 1,500 mg acetaminophen. - Fasting and postprandial (6 h) blood samples and VAS taken.	- WPI (17.6 g) pre-load.	- WPI (17.6 g) co-ingested.	- In individuals with T2D, glycaemia was greater throughout compared to healthy controls. - WP pre-load **↑** early insulin (~48% AUC_0−30min_) and GIP secretion (~15% AUC_0−30min_) without affecting PPG in both cohorts. - Rates of gastric emptying (i.e., acetaminophen pharmacokinetics) were ↔ between groups. - Gastric emptying was slower following the WP pre-load (*t* = 0–30 min). - WP timing did not affect postprandial lipemic markers in both groups.	PPG is similar when WP is consumed 15 min before or with a fat-rich meal, despite a delay in gastric emptying and an early increase in insulin following the WP pre-load.
Frid et al. (24)	Diet-controlled T2D (*n* = 14; 6F/8M) - Age: 27–69y - BMI: 26.2 ± 3.1 kg·m^2^ - HbA_1c_: 36 ± 3 mmol·mol - Dx: Not reported.	- Randomized, crossover design. - Two visits separated by 7d. - High GI breakfast and lunch (served 4 h post breakfast) served with addition either treatment or comparator. - Fasting and postprandial blood samples taken (4 h).	- WP (18.2 g)	- Ham and lactose (matched for protein and CHO).	- PPG ↔ between treatments at breakfast but was ↓ by 21% following lunch with whey ingestion. - WP ↑ insulin secretion by 53% and 49% (AUC_0−180min_) following breakfast and lunch, respectively. - WP ↑ GIP following both meals (20–34% AUC_0−180min_) with no effect on GLP-1.	The ingestion of WP at lunch reduces PPG, associated with an increase insulin and GIP concentrations.
King et al. (42)	Metformin ± diet controlled T2D (*n* = 11 M) - Age: 54.9 ± 2.3y - BMI: 31.8 ± 2.6 kg**·**m^2^ - HbA_1c_: 51.3 ± 3.4 mmol**·**mol - Dx: 4 ± 1y	- Randomized, single blind, crossover design. - Three visits separated by 7d. - Treatments were consumed immediately before mixed-nutrient breakfast (387 kcal: 56 g CHO) and lunch meals (879 kcal: 117 g CHO). - Fasting and postprandial (3 h) blood samples, and VAS taken. - Glycaemia monitored by CGM.	- WPC (15 g)	- WPH (15 g) - Null CON (water).	- WPC **↓** peak (~1.5 mmol·L) and PPG (~17% AUC_0−90min_) vs. CON. Glycaemia **↔** between WPC and WPC. - Total insulin secretion was **↑** (~14-20% AUC_0−180min_) following breakfast with WP treatments. - No supplemental effect on incretin peptides. - WP **↑** subjective feelings of satiety.	Consuming WP immediately before conventional breakfast and lunch meals reduces peak PPG and PPG excursions.
Jakubowicz et al. (31)	Metformin ± SU treated T2D (*n* = 15; 6F/9M) - Age: 64.1 ± 1.4y - BMI: 26.7 ± 1.2 kg·m^2^ - HbA_1c_: 50 ± 2 mmol·mol Dx: 8.0 ± 1.6y	- Randomized, crossover design. - Two visits separated by 14d. - Treatments consumed 30 min prior to a high-GI breakfast.Fasting and postprandial (3 h) blood samples taken.	- WPC (50 g)	- Null CON (water).	- WP ↓ peak PPG (~6 mmol·L) and total PPG (-28% AUC_0−180min_). - WP ↑ total insulin (+108% AUC_0−180min_), C-peptide (+43% AUC_0−180min_) and GLP-1 (+141% AUC_0−180min_) exposure. - WP ↑ intact/total GLP-1 ratio vs. CON (~65%) with ↔ on plasma DPP-IV.	Consuming a WP pre-load 30 min before a high-GI breakfast reduces peak PPG and PPG excursions, associated with increased secretion of insulin and GLP-1.
Ma et al. (19)	Diet-controlled T2D (*n* = 8; 1F/7M) - Age: 58 ± 3y - BMI: 28.6 ± 1.3 kg·m^2^ - HbA_1c_: 42 ± 2 mmol·mol - Dx: 5.4 ± 1.1y.	- Randomized, crossover design. - Three visits. - A soup pre-load consumed 30 min prior to a high-GI semi-solid meal (302 kcal: 59 g CHO). WP was either added to the pre-load, the meal or omitted. - Gastric emptying measured by scintigraphy. - Fasting and postprandial (6 h) blood samples taken.	- WP (55 g) in pre-load	- WP (55 g) in meal - Pre-load only (CON)	- WP ↓ peak glycaemia ~ 3 mmol·L vs. CON with ↔ between WP trials. - PPG (iAUC_0−300min_) ↓ following the WP pre-load (−51%) and when consumed with the meal (−45%); ↔ between WP trials. - WP pre-load ↓ early (0–45 min) PPG compared to WP in the meal. - ↑ Insulin, GLP-1, GIP and CCK responses with the WP pre-load. - Both WP trials delayed gastric emptying T50, which was the slowest with the WP pre-load (~35%).	Consuming WP 30 min before or with a high-GI breakfast reduces peak PPG and PPG excursions. WP is effective in stimulating insulin and incretin peptide secretion and delaying gastric emptying. A WP pre-load is more effective in reducing early PPG excursions compared to its consumption with a meal owing to an early increase in GLP-1 and slowing of gastric emptying.
Ma et al. (34)	Diet-controlled T2D (*n* = 7; 4F/3M) - Age: 60 ± 2y - BMI: 31 ± 2 kg·m^2^ - HbA_1c_: 42 ± 2 mmol·mol - Dx: not reported.	- Randomized, single-blind, crossover design.**Laboratory phase** - Days 1 and 28, treatments were consumed 30 min prior to a high-GI breakfast (314 kcal: 62 g CHO). - Gastric emptying measured by scintigraphy - Fasting and postprandial (6 h) blood samples taken.**Free-living phase** - Treatments consumed 30 min prior to each main meal for 28d before 14d washout.	- WPI (25g)	- Null CON (flavored beverage)	- Peak glycaemia was 1.3 mmol·L lower following WP vs. CON. - Gastric emptying was delayed after WP vs. CON. - No change in PPG or gastric emptying rates following 28d of supplement consumption. - Serum fructasomine tended to be lower following WP (*p* = 0.06) vs. CON. - HbA_1c_ unaffected by WP. - Energy intake and body mass similar throughout both 28d periods.	Consuming WP 30 min before a high-GI breakfast delays gastric emptying and reduces peak PPG.The ability of WP to regulate PPG and gastric emptying is sustained after its long-term consumption.
Mortensen et al. (48)	Metformin ± SU treated T2D (*n* = 12; 6F/6M) - Age: 64.6 ± 3.3y - BMI: 28.9 ± 3.7 kg·m^2^ - HbA_1c_: 51 ± 1 mmol·mol - Dx: not reported.	- Randomized, crossover design. - Four visits separated by 2–5wks. - Four different protein sources consumed with a high-fat test meal (~1,150 kcal: 45 g CHO). - Fasting and postprandial (8 h) blood samples taken.	- WP (45 g)	- Casein (45 g) - Cod (45 g) - Gluten (45 g)	- WP ↓ PPG (iAUC_0−480min_) by ~30–50% vs. to other treatments. - Insulin and incretin peptide responses were all similar between treatments. - WP ↓ postprandial Tg and free fatty acids (iAUC_0−360min_) by ~25% vs. other treatments.	Consumption of WP with a fat-rich meal reduces PPG excursions compared to casein, cod and gluten proteins, independent of increased insulin and incretin peptide concentrations.
Watson et al. (32)	Metformin ± diet-controlled T2D (*n* = 21; 5F/16M) - Age: 66 ± 2y - BMI: 30.8 ± 1 kg·m^2^ - HbA_1c_: 46.4 ± 1.5 mmol·mol - Dx: 6.3 ± 1.9y.	- Randomized, single-blind, crossover design. - Four visits separated by 4d. - Treatments (mixed with 150 ml water) consumed 15 min before semi-solid high-GI breakfast (369 kcal: 61 g CHO). - Gastric emptying measured by ^13^C GEBT. - Fasting and postprandial (4 h) blood samples taken.	- WP (17 g)	- Guar gum (5 g) - WP (17 g) + guar gum (5 g) - Null CON (sucralose; 60 mg)	- Early glycaemia (0–90 min) ↓ by 1-2mmol·L following WP and WP + guar gum vs. other treatments; ↔ between WP and WP + guar gum. - PPG (iAUC_−15−240*min*_) were ~15% lower following WP and WP + guar gum vs. other treatments; ↔ between WP and WP + guar gum. - WP ↑ insulin (~32%) and GLP-1 (~86%) iAUC_−15−240*min*_ vs. CON. - Both WP treatments delayed gastric emptying T50 vs. CON (~9–17%).	A WP pre-load, consumed 15 min before a high-GI meal, reduces PPG excursions compared to guar gum or a CON drink. WP is associated with an increase in insulin and GLP-1 and slowing of gastric emptying. Combining guar gum with WP did not further reduce PPG compared to WP.
Wu et al. (33)	Metformin-controlled T2D (*n* = 22 M) - Age: 64. 2 ± 1.4y - BMI: 27.9 ± 1.7 kg·m^2^ - HbA_1c_: 49 ± 2 mmol·mol - Dx: 5.6 ± 1.2y	- Randomized, double-blind, crossover design. - Four visits: VILD + whey; VILD + CON; placebo + whey; CON + placebo, separated by 7d. - Patients provided VILD (or PLA) 12 h and 1.5 h prior to high-GI MMTT. - Pre-loads (WP or CON) consumed 30 min prior to MMTT. - Gastric emptying measured by ^13^C GEBT. - Fasting and postprandial (4 h) blood samples taken.	- WPI (25 g)	- Null CON (water) - Vildagliptin (VILD; 50 mg) - Null placebo tablet	- Peak PPG ↓ following both WP + placebo and VILD + CON trial (~1 mmol·L); ↔ between WP + placebo and VILD + CON. - Greatest reductions in peak PPG seen following WP + VILD (~2.5 mmol·L). - WP + placebo delayed gastric emptying T50 vs. CON + placebo (~16%), which was further slowed with the addition of VILD (~31%). - WP + placebo ↑ total and intact GLP-1 and GIP, and insulin vs. CON + placebo (~10–120%). - Greatest incretin and insulin response seen following WP + VILD.	A WP pre-load reduces peak PPG, associated with delayed gastric emptying rates and increased insulin and incretin concentrations. WP produces comparable reductions in peak PPG as VILD. Combining WP with VILD has an additive effect on VILD's efficacy.

*A primary search of data was performed with search terms “whey protein,” “pre-load,” “pre-meal,” “second meal effect,” “postprandial glycaemia,” and “type 2 diabetes.” Studies were included if they involved adult participants with T2D, were acute laboratory-based studies with a randomized crossover design that included primary or secondary outcomes relating to PPG (i.e., AUC, iAUC, or peak glycaemia) following consumption of WP. The provision of WP of all commercially available forms (i.e., concentrate, isolate, and hydrolysed) were included when they were consumed in isolation from other dietary nutrients (i.e., dietary fibers)*.

*AUC, area under the curve; CGM, continuous glucose monitoring system; CON, control; DPP-IV, dipeptidyl-peptidase IV; Dx, duration of diabetes; GI, glycaemic index; GIP, glucose-dependent insulinotropic peptide; GLP-1, glucagon like peptide-1; iAUC, incremental area under the curve; MMTT, mixed-meal tolerance test; PPG, postprandial glycaemia; Tg, triglyceride; T50, gastric half-emptying time; VAS, visual analog scale; VILD, vildagliptin; WP, whey protein; WPC, whey protein concentrate; WPI, whey protein isolate; ^13^C GEBT, ^13^C gastric emptying breath test; ↓: reduced; ↔: similar; ↑: increased*.

To the authors' knowledge, only one study has directly compared mealtime WP with conventional pharmacological treatments (33). In an acute intervention involving 22 metformin-treated T2D males, Wu et al. (33) reported similar reductions in PPG between the provision of a WP (25 g) pre-load, consumed 30 min before a test meal, and administration of the DPP-IV inhibitor, vildagliptin. Remarkably, the authors showed that combining treatments enhanced vildagliptin's efficacy by two-fold compared to its sole administration (33). Whether the reported benefits are sustained long-term, or if they have any implications in the efficacy to treat T2D, remains unknown. Clearly, the strategy of combining a dietary and pharmacological approach is an attractive avenue that warrants further investigation.

## Current Limitations Impeding the Translation of Mealtime Whey Protein as a Therapy To Treat Type 2 Diabetes

Acute experimental trials show that mealtime WP can markedly improve PPG in people with T2D (Table 1) and may offer an adjunctive therapy for the treatment of this disease (33). However, investigations to date are largely focused on *proof-of-concept* rather than *efficacy to treat*, adopting methodological approaches that lack real-world applicability and may not portray what could be achievable in practice. For instance, studies have primarily examined the efficacy of mealtime WP to regulate PPG after the consumption of test meals that would seldom be consumed in the T2D community [i.e., powdered potatoes with glucose (19, 32–34); meals rich in saturated fat (47, 48)] and often over a single postprandial period (19, 31–34). The WP doses provided have also been unrealistically large (19, 31, 48) and presented in formats that do not align with contemporary living. While these studies prove effective in testing an experimental hypothesis, such approaches do not represent free-living eating behaviors and patterns. Therefore, it cannot be assumed that these results can be translated into everyday care or be effective in the long-term treatment of T2D.

## Implications for Future Research

### Supplemental Timing

The capacity of WP to regulate PPG is dependent on supplemental timing, specifically when consumed before- rather than with- (19) or following- (46) the main meal, pertaining to an early and pronounced secretion of GLP-1 and a slowing of gastric emptying (19). Yet, a significant caveat to this approach is the timing of the pre-load and commencement of the meal. For instance, a wealth of studies have presented WP 30 min before the nutrient challenge (19, 31, 33, 34, 44, 45), which is unlikely to replicate free-living behaviors. Indeed, adherence to prandial medications, which are prescribed to align with eating occasions, can fall short due to patient forgetfulness and the burden of having to plan ahead (49, 50). This similarly may be the case for WP with larger pre-load “windows.” Promisingly, WP consumed ≤ 15 min before a meal augment significant glycaemic benefit (32, 42, 46, 51, 52). There is additionally no further improvement in PPG when WP is consumed 30 min prior to- rather than 15 min before- the meal (51), which may have significance for patients adopting this strategy. However, the latter study was performed in individuals with the Metabolic Syndrome and not those with overt diabetes. It is unclear if such findings are translatable to T2D individuals.

### Whey Protein Dose

Evidence to date has primarily presented unrealistically large WP doses (25–55 g), entailing a significant caloric load with a large financial cost associated (19, 31, 33, 48). Indeed, the glucose-lowering efficacy of WP is reported to be dose-dependent (44). However, when accounting for the energy content of WP pre-loads (20–70 g), despite reducing energy intake at *ad libitum* meals compared to a control treatment, cumulative energy intake is similar (44, 53). This may explain why there were no differences in body mass in overweight and obese individuals with (34) and without (54) T2D following long-term (4–12wks) mealtime WP supplementation [54–75 g·d^−1^ (250–280 kcal·d^−1^)]. Given the importance of weight management in the care and prevention of T2D (12), and where higher out-of-pocket costs are associated with poor adherence to anti-diabetic treatments (50), this needs careful consideration when designing long-term trials or assessing current evidence. Promisingly, small amounts of mealtime WP (<100 kcal) have been shown to reduce PPG in people with T2D (32–34, 42) and are reported to not cause weight-gain following their sustained consumption (34). These data suggest that the consumption of mealtime WP (<100 kcal) may modulate daily energy intake, which could be a secondary mechanism to improve PPG through a reduction in meal size. However, to the best of the authors' knowledge, the satiating effects of mealtime WP of any amounts has not been assessed in individuals with T2D.

### Long-Term Efficacy

Investigations examining the efficacy of mealtime WP beyond single isolated meals are few in number (*n* = 2; Table 1), which is surprising given postprandial hyperglycaemic excursions display significant diurnal variances (55–57) and are highly prevalent throughout the day (13). Promisingly, we have demonstrated the capacity of mealtime WP to regulate PPG excursions beyond a single meal in adult males with T2D (42). In an acute, randomized-control trial, consuming WP (15 g) immediately prior to conventional breakfast and lunch meals reduced PPG excursions (~17% AUC_0−90min_) compared to an energy-free control beverage (42). Although exploratory in nature, these findings may have importance considering glycaemic excursions following breakfast and lunch markedly contribute to the prevalence of daytime hyperglycaemia in T2D patients (13). Whether such benefits are sustained long-term and under free-living conditions, or if they have any implications in the treatment of T2D, is unclear.

To the best of the authors' knowledge, only one investigation has examined the long-term efficacy of mealtime WP to treat T2D. In a randomized-control trial, Watson et al. (52) reported statistically significant, but arguably clinically insignificant, improvements in HbA_1c_ (−1.0 mmol·mol) following 12wk consumption of a pre-meal WP *shake* compared to a void comparator. However, this study was not designed to ascertain the relative influence of each compound of the pre-load [WP (17 g) or guar gum (5g)], which may both individually regulate glycaemia (32). The studied T2D cohort also had excellent glycaemic control at baseline (~49 mmol·mol) contributing to the very modest reductions in HbA_1c_ (58). Conversely, in a pilot study involving seven well-controlled people with T2D, serum fructosamine, which reflects recent glycaemic control, *tended* to be lower (*p* = 0.06) following a 28d period of pre-meal WP (25 g) compared to an inert placebo (34). While the above studies suggest that mealtime WP may positively affect long-term glycaemic control (34, 52), this is vastly under-researched and requires validation in future trials.

Nonetheless, evidence from the latter two studies suggests that the pancreatic β-cells and gluco-regulatory mechanisms within the small intestine retain their sensitivity to prolonged WP exposure (34, 52). This is important considering GLP-1-mediated regulation of gastric emptying and PPG are subjected to rapid tachyphylaxis following the incretin peptide's sustained exposure (59, 60), where adaptive changes by which nutrients regulate gastrointestinal function have also been reported following prolonged intake of fat (61) and glucose (62).

### Behavioral Change or the Hawthorne Effect?

The Hawthorne effect describes when an individual alters their behavior in response to being observed, often in a positive manner (63), making it increasingly problematic to disentangle the efficacy of an intervention to treat long-term glycaemic outcomes (64). Indeed, the effectiveness of dietary interventions when unassisted can be poor (65). Accordingly, this needs consideration when critiquing current evidence and determining mealtime WP's sustainability out with of the controlled research environment. It is therefore essential to adopt a multi-dimensional approach; assessing not only quantitative outcomes but also highlighting patient behaviors, and barriers and facilitators to this strategy. Indeed, such approaches are fundamental aspects of successful diabetes care (12).

### Free-Living Glycaemic Variability

One study conducted by Watson et al. (52) suggested that mealtime WP *might* improve free-living PPG control as hypothesized from the observed reductions in HbA_1c_. However, meeting clinically desirable HbA_1c_ values does not guarantee negligible postprandial hyperglycaemic excursions (13). Indeed, reliance on HbA_1c_ to characterize alterations in glycaemic control fails to account for daily glycaemic excursions and variances (66).

In an era of personalized and precision medicine, advancements in continuous glucose monitoring systems [CGM] can overcome limitations associated with surrogate measures of glycaemia (67). CGM allows for the detailed assessment of intra- and inter-daily glycaemic excursions under free-living conditions (66, 67). Yet, there is limited information regarding 24 h glycaemic exposure following concerted dietary interventions.

One small study has used CGM to assess free-living glycaemic control following mealtime WP (21 g) in people with T2D over a 48 h free-living period, reporting no benefit of WP compared to an inert placebo (68). However, such findings were largely compounded by the heterogenic cohort studied, as acknowledged by the authors, and may have been affected by various extraneous variables that were not accounted for. The 48 h period assessed is substantially less than what is recommended when assessing free-living glycaemia with CGM (67)—albeit such recommendations are primary care specific. Nonetheless, duration of assessment needs consideration for future investigations, particularly when free-living behaviors are known to change when under observation (63).

### Homogenous Study Populations

Previous interventions have studied the application of mealtime WP in people with well-controlled T2D (HbA_1c_ <53 mmol·mol) treated with first-line therapies, excluding individuals with less stringent glycaemic control and treated with intensified regimens. Indeed, individuals *likely* to see the greatest benefit of mealtime WP are those with moderately-controlled diabetes (HbA_1c_ <70 mmol·mol) where PPG is the predominant contributor to overall glycaemic control (1). Yet, considering that at a population level only ~50% of people with T2D meet the HbA_1c_ target of ≤ 53 mmol·mol (69), and within 6y of starting oral hyperglycaemic agents, ~25% require exogenous insulin (70), evidence is based on cohorts that are not representative of the wider patient population.

The regulation of PPG also occurs, in part, by insulin-independent mechanisms (37, 40, 71–73). Thus, for individuals with more advanced diabetes, the use of strategies that modulate PPG by pancreatic and extra-pancreatic means is appealing (40). It is conceivable that the pleiotropic constituents and stimulated pathways following mealtime WP (Figure 1) may offer clinical benefit to individuals with limited β-cell function or advanced T2D, who presently have been ignored when studying WP's glucose-lowering efficacy.

### Combination Therapy

T2D is largely defined with *relative* rather than *absolute* insulin deficiency (74). Therefore, strategies that enhance β-cell activity may be of therapeutic benefit for those with advanced diabetes. Indeed, GLP-1 receptor agonists work, in part, by enhancing β-cell activity (75). Given the incretin and insulinotropic properties of WP purported to regulate PPG (Figure 1), combining WP with basal therapies may present an effective long-term strategy to enhance glycaemic control. This concept has proven effective with the combination of basal insulin and incretin agonists compared to basal insulin regimens alone in poorly-controlled T2D patients (76, 77). With this in mind and where WP enhances vildagliptin's efficacy (33), combining mealtime WP with anti-diabetic agents may present a novel strategy to achieve glycaemic control coupled with the low propensity of adverse side effects such as weight gain and hypoglycaemia.

### The Presentation of Whey Protein and Its Associated Implications for Free-Living Adherence

Adherence to anti-hyperglycaemic treatments is not a single behavior but a dynamic constellation of behaviors influenced by social, environmental and individual circumstances (69). Thus, understanding the various psychosocial elements associated with a given treatment is critical to maximizing its application and clinical utility (12). To the best of the authors' knowledge, these are factors that have not been considered with the application of mealtime WP.

Studies have presented WP as dry powders that require dilution with water, producing solutions that are often inconvenient and large in volume (~350 ml) (19, 31) and largely unpalatable, requiring further flavoring (19, 31, 33) and “mouth rinsing” to eliminate any aftertaste (46, 78). Although effective under controlled conditions, this unlikely represents a convenient approach outside of the laboratory. Indeed, there is a general unwillingness to consume powdered protein supplements publicly (79) with taste and convenience largely determining eating behaviors (80). Even where available evidence suggests the use of a particular treatment, patient preference determines their application (12). Thus, to ensure free-living applicability and long-term sustainability, the pre-loads provided need to be extrapolated into treatments that are compatible with contemporary lifestyles. Given the preponderance and social acceptance of “grab and go” foods, providing a palatable and discrete WP beverage, similar to a yogurt drink for example, may be an effective strategy to increase adherence and exert clinical benefit within the community setting.

### Potential Adverse Effects

When considering the therapeutic use of mealtime WP, it is also pertinent to recognize potential adverse events that may occur. Indeed, concerns have been raised as a consequence of elevated nutrient signaling that may accelerate pancreatic islet fatigue and failure. Primarily, these are observations from incidence rates of diabetes in pre-diabetic mice models fed a high-protein diet (81, 82). However, there are also reductions in the efficacy of oral insulin secretagogue therapies over time–in part due to a decline in the β-cells' insulin-secretory capacity (83)–where their use are also reported predictors of exogenous insulin initiation (84).

Long-term exposure of hyperinsulinemia is reported to compromise whole-body insulin sensitivity (85) and increase the atherosclerotic milieu (86). Therefore, the chronic application of WP and its associated insulinotropic effects may counterintuitively desensitize insulin's action and be associated with adverse cardio-metabolic outcomes. However, the long-term administration of sulfonylureas', which increase both basal and postprandial insulinaemia, are not associated with an increase in cardiovascular events (6). Furthermore, the periodic elevations in postprandial insulinemia that follow WP ingestion (~20 g) persist for ~120 min (32, 33, 87), where sustained WP supplementation is associated with a reduction, rather than an increase, in metabolic risk factors in insulin-resistant individuals (54, 88, 89). These data suggest that the insulinotropic properties of WP would pose little consequence to long-term metabolic health. Yet, mealtime WP's influence on the regulation of hepatic lipid metabolism, which is sensitive to hyperinsulinemia and is upregulated in insulin-resistant states (90), is not known. Considering the reciprocal relationship between hyperinsulinemia and steatosis (91), dyslipidaemia (92), and cardiovascular events (93), this is an avenue that needs further consideration.

Ambient elevations in fasting plasma BCAA are a metabolic signature of dysglycaemic populations (94–96), and prospectively predict T2D (97, 98), suggesting that dietary protein/BCAA intake could be an important regulator of glucose tolerance. Indeed, BCAA surfeit through infusion/pulse feeding protocols impairs insulin signaling and glucose disposal in humans (99–101). However, the observed elevations in plasma BCAA within insulin-resistant individuals may be attributed to dysregulated BCAA catabolism (102) rather than dietary BCAA intake *per se* (103, 104). Moreover, and in contrast to BCAA infusion/pulse feeding protocols, the surge in plasma BCAA following protein consumption are not sustained, returning to their nadir within ~180 min (42, 105). In fact, the acute influx of amino acids following dietary protein pre-loads are associated with increased β-cell sensitivity to glucose (106) and improved PPG handling (105), as has been demonstrated acutely across various insulin-resistant populations (19, 31–33, 42, 46, 107). It, therefore, appears unlikely that the BCAA content of a small WP pre-load would be sufficient to exacerbate defects in insulin sensitivity as observed under BCAA surfeit (99–101).

Dietary proteins, in addition to stimulating insulin secretion, also stimulate the release of glucagon from pancreatic alpha-cells (108), which initiates hepatic glucose output. Indeed, several studies demonstrate that mealtime WP supplementation increases postprandial glucagonemia in insulin-resistant individuals with (32, 33) and without (51, 87, 109) overt T2D. However, despite increasing glucagon concentrations, PPG is consistently improved following the ingestion of WP (32, 33, 51, 87, 109). This is likely due to the concomitant increase in insulin concentrations, which is particularly effective in suppressing glycogenolysis (108). The consumption of WP also increases GLP-1 (19, 31–33), which may suppress hepatic glucose output (110) and enhance peripheral glucose uptake (72), independent of changes in islet hormones. Nonetheless, the relevance of increasing glucagon concentrations following consumption of mealtime WP in relation to PPG remains controversial. Future studies should include approaches designed to assess mealtime WP's influence on the relative contribution of glucose from both exogenous and endogenous origins to the total glucose pool. This would also provide a greater understanding of the mechanisms underpinning WP's gluco-regulatory effects.

## Future Research Considerations

### Clinically Important Outcomes

Acute studies demonstrate immediate glycaemic benefit from mealtime WP, providing valuable information on the immediate post-meal responses. However, whether such outcomes translate to clinically meaningful benefits are unclear. Indeed, changes in metabolic health require chronic improvements in PPG and other cardio-metabolic markers, outcomes that cannot be achieved within the acute setting.

One study has investigated the role of pre-meal WP to treat T2D, as quantified by HbA_1c_ (52). While HbA_1c_ is used as a primary outcome to assess glycaemic control and is a surrogate marker assessing the risk of diabetes complications (4), it fails to tell researchers or clinicians other meaningful outcomes outside of the weighted-average glycaemia over the preceding ~12wks (66). Nonetheless, the importance of reporting HbA_1c_ for the appraisal of diabetic treatments should not be undervalued; rather, it should be complimented by other glycaemic measures (67). Studies are needed to include and specify further clinically meaningful glycaemic outcomes that not only include HbA_1c_ but also time in euglycaemic range, frequency and severity of hypoglycaemic and hyperglycaemic events, glycaemic variability, and patient-reported outcomes.

### Understanding the Clinical Potential in the Wider Diabetes Population

Investigations have studied the efficacy of mealtime WP in patients that do not represent the wider T2D population, excluding those with more advanced diabetes and treated with insulin regimens. Yet, insulin-dependent T2D individuals retain varying degrees of clinically functioning β-cells (74) and might be responsive to nutrient-mediated β-cell stimulation. The complex and pleiotropic actions of mealtime WP (Figure 1) may also present therapeutic benefit to individuals with limited β-cell function (40). Studies are encouraged to investigate this therapy within realistic T2D populations and to adopt approaches to disentangle its mechanisms to treat hyperglycaemia.

### Design of Studies

To examine the real-world sustainability of mealtime WP, studies are encouraged to provide WP beverages that are in formats and at times that complement modern living patterns. It is also essential for future investigations to work with the patient, highlighting potential barriers and facilitators to the application of this mealtime strategy.

### Supplemental Dose

It is important to consider the additional energy associated with mealtime WP when evaluating its long-term application. Although work demonstrates that the consumption of 15–20 g (~100 kcal) of WP improves PPG in T2D individuals (32, 42), this may translate to an increase in long-term gross energy intake. The minimum efficacious dose of mealtime WP needs to be established.

## Conclusion

For people with controlled T2D, experimental evidence demonstrates that the application of pre-meal WP results in acute improvements in PPG, which if sustained long-term, may have therapeutic significance. Yet, the clinical application of mealtime WP remains to be established with literature to date idling on proof of concept rather than efficacy to treat T2D. Addressing the experimental limitations highlighted in this report are critical to understanding the mechanisms underpinning WP's potential clinical benefit and assessing its acceptance within the wider scientific community. Only then can mealtime WP be considered a therapeutic option for people with T2D.

## Author Contributions

KS, KB, ES, and DW wrote and reviewed the manuscript. All authors approved the final manuscript.

## Conflict of Interest

DW and ES have received research funding from Arla Foods Ingredients (AFI), Viby J, Denmark. DW and ES have received travel expenses and consultancy fees from AFI. ES has received research funding from The Dairy Council. This review was written independently from industrial funders. The remaining authors declare that the research was conducted in the absence of any commercial or financial relationships that could be construed as a potential conflict of interest.

## Abbreviations

BCAA: branched chained amino acids
CGM: continuous glucose monitoring system
DPP-IV: dipeptidyl peptidase IV
GLP-1: glucagon-like peptide 1
PPG: postprandial glycaemia
T2D: type 2 diabetes
WP: whey protein.
